# Probing the structure and function of locus coeruleus projections to CNS motor centers

**DOI:** 10.3389/fncir.2022.895481

**Published:** 2022-09-29

**Authors:** Barry D. Waterhouse, Haven K. Predale, Nicholas W. Plummer, Patricia Jensen, Daniel J. Chandler

**Affiliations:** ^1^Department of Cell Biology and Neuroscience, Rowan University, Stratford, NJ, United States; ^2^Neurobiology Laboratory, Department of Health and Human Services, National Institute of Environmental Health Sciences, National Institutes of Health, Raleigh, NC, United States

**Keywords:** locus coeruleus, norepinephrine, motor centers, viral vector track tracing, TrAC

## Abstract

The brainstem nucleus locus coeruleus (LC) sends projections to the forebrain, brainstem, cerebellum and spinal cord and is a source of the neurotransmitter norepinephrine (NE) in these areas. For more than 50 years, LC was considered to be homogeneous in structure and function such that NE would be released uniformly and act simultaneously on the cells and circuits that receive LC projections. However, recent studies have provided evidence that LC is modular in design, with segregated output channels and the potential for differential release and action of NE in its projection fields. These new findings have prompted a radical shift in our thinking about LC operations and demand revision of theoretical constructs regarding impact of the LC-NE system on behavioral outcomes in health and disease. Within this context, a major gap in our knowledge is the relationship between the LC-NE system and CNS motor control centers. While we know much about the organization of the LC-NE system with respect to sensory and cognitive circuitries and the impact of LC output on sensory guided behaviors and executive function, much less is known about the role of the LC-NE pathway in motor network operations and movement control. As a starting point for closing this gap in understanding, we propose using an intersectional recombinase-based viral-genetic strategy TrAC (Tracing Axon Collaterals) as well as established *ex vivo* electrophysiological assays to characterize efferent connectivity and physiological attributes of mouse LC-motor network projection neurons. The novel hypothesis to be tested is that LC cells with projections to CNS motor centers are scattered throughout the rostral-caudal extent of the nucleus but collectively display a common set of electrophysiological properties. Additionally, we expect to find these LC projection neurons maintain an organized network of axon collaterals capable of supporting selective, synchronous release of NE in motor circuitries for the purpose of coordinately regulating operations across networks that are responsible for balance and movement dynamics. Investigation of this hypothesis will advance our knowledge of the role of the LC-NE system in motor control and provide a basis for treating movement disorders resulting from disease, injury, or normal aging.

## Overview

The goal of this report is to identify major unanswered questions regarding locus coeruleus (LC) regulation of signal processing in motor control circuits of the mammalian brain and to propose a methodology for pursuing these questions. This path of inquiry is an extension of recent studies that challenge the conventional view of the LC as a broadly projecting, homogeneous cluster of norepinephrine (NE)-containing cells with little input or output specificity. The revised hypothesis is that LC maintains an intrinsic organization which respects the cognitive, sensory, and motor functions of its efferent targets. Here we postulate that this ordered structure extends to the relationship between LC and supraspinal motor networks. A closer examination of the organization of LC connections to motor centers in the brain will provide a platform for determining the consequences of activation or suppression of LC-motor circuit projections on motor behavior in normal animals and animals whose movements are compromised by disease, injury, or aging.

Locus coeruleus was first identified in the human brainstem by J. Reil more than 200 years ago (Reil, 1809). Over a century later histochemical (Carlsson et al., 1962; Dahlström and Fuxe, 1964), immunohistochemical, and autoradiographic (Jones et al., 1977; Jones and Moore, 1977) techniques were used to identify NE as the small molecule transmitter synthesized in all LC neurons and to establish the broad projection of LC axons throughout the forebrain, brainstem, cerebellum, and spinal cord of all mammalian species. Since these early studies there has been sustained interest in determining the role of the LC-NE system in brain function and behavior. Initially, it was thought that this tightly clustered group of NE-containing cells was a rostral extension of the sympathetic chain and that its role, like its counterparts in the periphery, was to release NE broadly and indiscriminately throughout the CNS as an “alarm” signal (Grant et al., 1988) in response to “fight or flight” conditions. Currently, a far more complex view of the role of the system in brain function and behavior has emerged; one that posits LC regulation of behavioral state and state dependent signal processing (Berridge and Waterhouse, 2003) by way of NE-mediated neuromodulatory actions on cells, circuits, and neural networks within the CNS. Most of this work has focused on LC-NE modulation of sensory (Waterhouse et al., 1990; Bouret and Sara, 2002; Devilbiss and Waterhouse, 2004; Devilbiss et al., 2006; Linster et al., 2011; Martins and Froemke, 2015; Hirschberg et al., 2017; Kane et al., 2017; Navarra et al., 2017; Glennon et al., 2019), affective (McCall et al., 2015; Borodovitsyna et al., 2020) and cognitive (Bouret and Sara, 2004; Sara and Bouret, 2012; Cope et al., 2019; Bari et al., 2020; Carlson et al., 2021) circuit operations, with almost no attention paid to the impact of the LC-NE system on motor circuits, motor networks, and movement generation.

The potential for this system to regulate motor function according to the behavioral demands of the organism is significant (Freedman et al., 1977; Moises et al., 1979, 1980, 1981, 1983; Moises and Woodward, 1980; Watson and McElligott, 1983, 1984; Bickford et al., 1985, 1992, 1999; Bickford, 1993; Nelson et al., 1997; Clayton et al., 2004). For example, early work in anesthetized rat showed that local NE administration *via* microiontophoresis or electrical stimulation of LC could augment cerebellar Purkinje cell responses to putative amino acid transmitters and afferent synaptic inputs while not having a direct effect on spontaneous firing rate (Freedman et al., 1977; Moises et al., 1979, 1981, 1983; Moises and Woodward, 1980). The facilitating actions of NE on Purkinje cell responses to GABA were further shown to be the result of beta receptor activation and cyclic AMP-mediated regulation of GABA A receptor function (Sessler et al., 1989; Cheun and Yeh, 1992, 1996). Also in cerebellum, Woodward and colleagues demonstrated the ability of locally applied NE to gate visual input signals to Purkinje cells that were not initially responsive to these inputs and at higher doses to sharpen the visual receptive fields of these cells (Woodward et al., 1991b). Collectively these studies revealed neuromodulatory actions of NE and LC activation at the cellular level but left open the questions of how such effects could influence: (1) the responses of ensembles of Purkinje neurons to sensorimotor inputs, (2) the responses of the cerebellar circuitry as-a-whole to such information, and (3) the contributions of output from the cerebellum to motor behavior as LC activity waxes and wanes across the waking state.

The holistic impact of LC output and NE release on cerebellar operations, specifically, and supraspinal motor centers, in general, remains speculative at this time given our current limited understanding of the anatomy and physiology of LC projections to motor centers. For example, under conditions requiring rapid, forceful yet precise movement, output from the LC-NE system may be required to optimize operations at cellular, circuit and network levels, thereby improving the flow of information through the motor network and ultimately facilitating the execution of reflexive and goal-directed motor behavior. Ample evidence also implicates the LC-NE system in motor dysfunction (Feeney et al., 1993; Von Coelln et al., 2004; Rommelfanger et al., 2007; Rommelfanger and Weinshenker, 2007; Vazey and Aston-Jones, 2012; Liu et al., 2013; Osier and Dixon, 2016; Vermeiren and Deyn, 2017; McPherson et al., 2018; Sternberg and Schaller, 2019; Yin et al., 2021). Nevertheless, the details of LC-interactions with CNS motor centers and subsequent influences on motor behavior are lacking. In the context of the new view of the modular design of the LC-NE system, our contention is that segregated channels of LC output coordinately regulate neuronal responsiveness and signal transfer in motor centers across the brain, thereby improving motor neuron, motor circuit and motor network operations and ultimately leading to improved motor function as behavioral circumstances dictate. Such actions would parallel the recently demonstrated facilitating effects of NE on signal processing and performance of sensory guided behaviors [see review – (Waterhouse and Navarra, 2019)]. It is important to note that segregated operation of the LC-NE system does not preclude a mode of operation where inputs to LC drive output to all its terminal fields simultaneously such as might occur in the transition from sleep to waking and generalized arousal.

**FIGURE 1:**
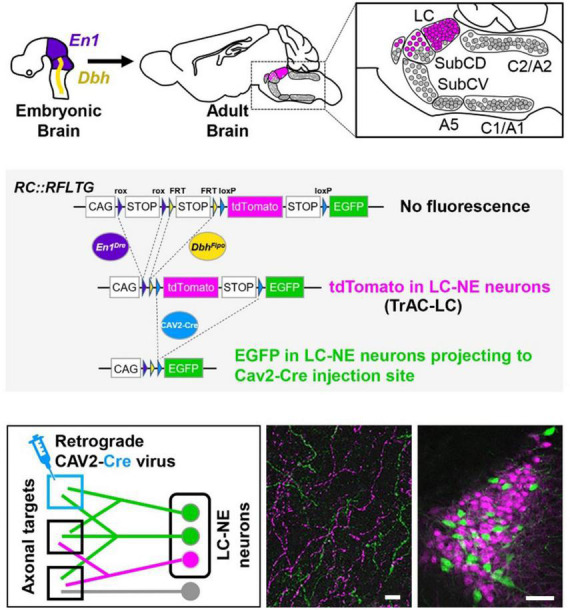
TrAC (Tracing Axon Collaterals) permits fluorescent labeling of genetically defined neuron populations based on axonal projections. **(Top)** Neurons with a history of *En1* and *Dbh* expression constitute the locus coeruleus and an adjacent portion of dorsal subcoeruleus. **(Center)** In mice heterozygous for *En1^Dre^*, *Dbh^Flpo^*, and the *RC:RFLTG* indicator allele, locus coeruleus (LC)-norepinephrine (NE) neurons are labeled constitutively with tdTomato (red fluorescence) and switch to EGFP (green fluorescence) after Cre recombination. **(Bottom)** After injection of a retrograde CAV2-Cre virus **(left schematic)**, EGFP labels LC-NE neurons projecting to the injection site **(right image)** as well as all their axon collaterals in other brain regions **(middle image)**, scale bars = 100 μm. Adapted from Plummer et al. (2020).

To address the above issue a recently developed viral-genetic method TrAC (Tracing Axon Collaterals) (Plummer et al., 2020) is available to examine, in detail, the structural features and physiological attributes of LC-NE neurons that project to specific motor centers in the rodent brain (see Figure 1). This approach allows for determination of the full extent of the distribution of axon collaterals from LC cells to motor centers and non-motor centers throughout the CNS. As such, a major goal going forward is to address a larger issue which has been the subject of considerable speculation for many years but, until now, has eluded rigorous experimental testing, i.e., *are axonal projections from LC cells ubiquitously and non-selectively distributed across the brain and spinal cord or are they organized according to the functional properties of their efferent targets*. In addition, using TrAC the soma and dendritic arbors of LC projection cells are revealed. Thus, the intranuclear distribution of LC-motor network projection cells and the distribution of their dendritic fields in the peri-coerulear space where afferent inputs arrive, can be examined. In addition, *ex vivo* electrophysiology can be employed to determine the physiological properties of these cells. Overall, this combined experimental strategy has the potential to provide new information about how it may be possible for the LC-NE system to coordinately regulate activity in neural circuits that are modality specific but broadly distributed across the CNS. Additional work in behaving animals can be designed to assess how output from LC affects performance in motor tasks that require coordination, balance, and manual dexterity.

## New theoretical framework for evaluating locus coeruleus influences on motor network operations

Our laboratory and many others [see reviews – (Woodward et al., 1991a; Berridge and Waterhouse, 2003; Aston-Jones and Cohen, 2005a; Uematsu et al., 2017; Waterhouse and Navarra, 2019)] have established a neuromodulatory role for the LC-NE system such that LC activation and NE release promote enhancement of individual and ensemble neuronal responses to afferent inputs, thereby facilitating signal transfer through neural circuits and neural networks that receive LC projections. Based on prior anatomical and physiological data such effects have been presumed to occur uniformly and simultaneously throughout all LC terminal fields as LC output rises and falls across different behavioral states. New data argue for a more heterogeneous, modular organization and operation of LC (Chandler and Waterhouse, 2012; Chandler et al., 2014a,^2019^; Hirschberg et al., 2017; Uematsu et al., 2017; Poe et al., 2020). In light of these findings four major questions have emerged regarding the structure and physiology of the LC efferent pathway. ***First***, are LC axon collateral networks organized according to terminal field function? The nucleus gives rise to a brain-wide network of NE-containing fibers; however, the branching patterns and distribution of LC axons have not been elucidated for any brain region. ***Second***, are the dendritic arbors of LC cells with preferred projection targets distributed within discrete sub-regions of the peri-coerulear space? Prior studies in rodents have demonstrated a topographic ordering of inputs to the peri-coerulear space indicating the potential for selective afferent regulation of subsets of LC cells whose dendrites populate this space. The question here is whether LC cells projecting to motor centers of the forebrain exhibit a preferred peri-coerulear distribution of their dendrites, thereby positioning them to receive inputs from specified afferent pathways. ***Third***, do subsets of LC neurons with preferred projection targets exhibit unique electrophysiological properties and excitability thresholds such that differential, asynchronous release of NE across different LC terminal fields is possible? ***Fourth***, how does activation or inactivation of sub-populations of modality-specific LC cells impact performance of cognitive, sensory, or motor behavioral tasks? While some information is available regarding the anatomical organization and electrophysiological characterization of sub-sets of LC cells that project in a coordinated fashion to cognitive and sensory regions of the spinal cord and forebrain (Simpson et al., 1997; Chandler et al., 2014a,b; Hirschberg et al., 2017; Wagner-Altendorf et al., 2019), the relationship between the LC-NE system and motor circuits of the mammalian brain has largely been ignored. The absence of this information prevents us from gaining a full appreciation for the role of the LC-NE system in motor control and movement. In a more global sense the question is, what anatomical and physiological features of LC neurons allow for specificity or, alternatively, uniformity of action across broadly distributed regions of the CNS that receive projections from the LC-NE system? A more complete understanding of the anatomical organization and physiological attributes of LC efferent projections to motor networks would greatly benefit theoretical constructs of LC-NE influences on behavioral outcomes, in general, and motor functions, in particular.

## Outstanding questions and experimental strategies

### Do locus coeruleus efferent projections maintain a unique relationship with motor-related terminal fields through distribution of a functionally ordered network of axon collaterals?

Early studies revealed moderate to dense distributions of NE-containing fibers in rodent primary and secondary motor cortices, motor thalamus, brainstem motor centers and cerebellum (Bloom et al., 1971, 1972; Siggins et al., 1971; Hoffer et al., 1973; Lindvall et al., 1974; Levitt and Moore, 1978, 1979; Morrison et al., 1978; Nelson et al., 1997). The LC was identified as a major source of these fibers (Siggins et al., 1971; Hoffer et al., 1973; Steindler, 1981). Many early demonstrations of LC-NE modulatory effects on synaptic transmission and neuronal responsiveness to putative transmitter application were carried out in cerebellum (Freedman et al., 1977; Moises et al., 1979, 1983; Moises and Woodward, 1980). Later this work was extended to sensory circuits throughout the brain and hippocampus (Segal and Bloom, 1974a,b, 1976a,1976b; Waterhouse et al., 1980, 1982, 1986, 1990; Kasamatsu and Heggelund, 1982; Holdefer and Jacobs, 1994; Manunta and Edeline, 1997; Waterhouse and Navarra, 2019). It is now well established that output from the LC-NE system can regulate sensory signal processing; i.e., increased neuronal responsiveness to sensory-driven inputs, altered feature extraction properties of sensory neurons, and facilitation of the transfer of sensory information through ascending sensory networks (Rogawski and Aghajanian, 1980a,b; Waterhouse et al., 1990; McLean and Waterhouse, 1994; Devilbiss and Waterhouse, 2004, 2011; Devilbiss et al., 2006). More recent studies show that LC-NE actions at the level of individual cells and local circuits can influence the outcome of sensory guided behaviors (Jiang et al., 1996; Escanilla et al., 2010; Linster et al., 2011; Martins and Froemke, 2015; Navarra et al., 2017) and prefrontal cortex dependent executive functions (Arnsten and Li, 2005; Berridge et al., 2006, 2011; McGaughy et al., 2008; Berridge and Devilbiss, 2011; Chandler et al., 2014b; Spencer et al., 2015; Cope et al., 2019; Glennon et al., 2019). However, despite longstanding evidence of LC-NE innervation across motor regions of the brain, there is a major gap in our understanding of how NE release affects signal processing in these circuits and ultimately exerts control over motor activities. This gap is even wider when considered relative to our increasingly sophisticated understanding of the influence of the LC-NE system on sensory processing and executive function. Determination of the anatomical relationships between LC and supraspinal motor centers in rodent brain is a critical first step in closing this gap.

Knowledge of the input-output relationships of LC is essential to postulating the spatial and temporal implications of LC-mediated release of NE in LC innervated motor networks. Motor control is a complex, multi-dimensional process, achieved through integration of activity across a broadly distributed network of brain and spinal cord circuits (Brooks, 1986, The Neural Basis of Motor Control, Oxford Press). Although nearly all CNS movement-related areas are innervated by LC, the projections to primary motor cortex, motor thalamus (VL nuc), red nucleus, lateral vestibular nucleus, cerebellar cortex and deep cerebellar nuclei (lateral, intermediate, medial) should be the initial focus of inquiry as these regions are interconnected through well-established input-output pathways and are engaged in maintaining balance; planning, execution and coordination of voluntary movements; and postural adjustments during movement sequences (see Figure 13.2 – Brooks, 1986, The Neural Basis of Motor Control Oxford Press). One could imagine that simultaneous NE release across motor circuits in the forebrain *via* a topographically ordered network of axon collaterals from motor selective LC neurons would result in coordinated noradrenergic modulation of neural operations dedicated to an ongoing motor directive. We propose TrAC as the means to advance this idea. In brief, a transgenic line of mice has been developed whereby LC-NE neurons constitutively express tdTomato fluorescent label throughout their soma, dendrites, and axonal arbors (Robertson et al., 2013; Plummer et al., 2015, 2020). These cells can be selectively switched to expression of EGFP following unilateral injections of the CAV2-Cre canine adenoviral vector in targeted regions of the central motor network, thus revealing the sub-population of LC cells with specified projections to the injection site (Hnasko et al., 2006).

**FIGURE 2:**
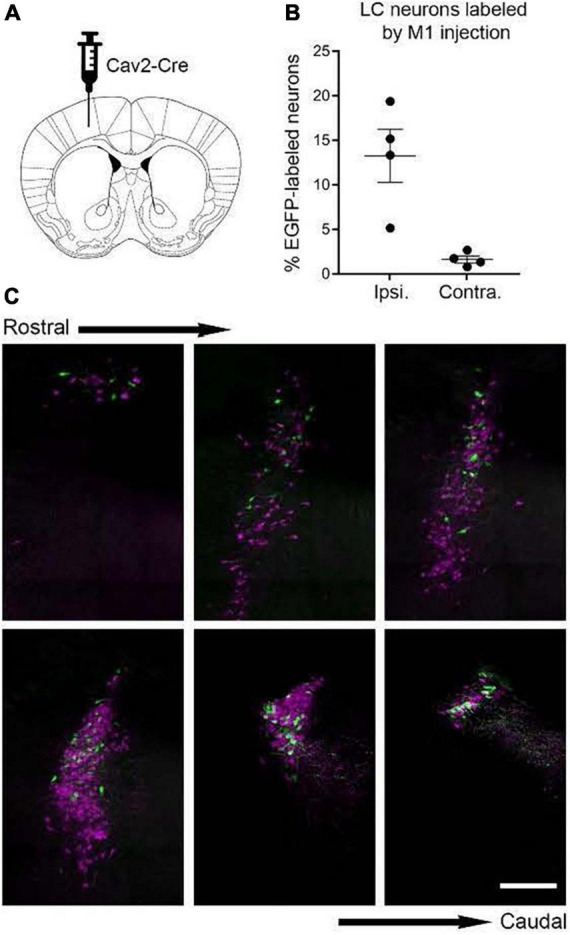
Labeling of locus coeruleus neurons in TrAC-LC mice following CAV2-Cre injection in primary motor cortex (M1). **(A)** Coronal schematic of mouse forebrain section showing position of CAV2-Cre injection. **(B)** Bar graph showing percentage of EGFP-labeled LC neurons, ipsilateral and contralateral relative to the injection site (*n* = 4 mice). **(C)** Representative coronal sections through the rostrocaudal extent of the ipsilateral LC showing distribution of EGFP-labeled (green) and tdTomato-labeled (magenta) cells. Scale bar, 200 μm. Adapted from Plummer et al. (2020).

After viral vector injections in any one of the areas of interest, retrogradely transduced cells in LC will express EGFP in their soma and throughout their axonal trees thus revealing the distribution of axon collaterals from those cells to all potential terminal fields in the brain including the site targeted for injection. Experiments of this nature would aid in determining the distribution and pattern of axon collateralization from LC cells that have defined motor circuit projections. Axonal networks associated with injections in thalamic and cortical visual terminal fields would serve as controls for comparison. In preliminary studies using TrAC we found labeled primary motor cortex-projecting NE neurons (EGFP +) intermingled among tdTomato + neurons within the ipsi- (Figures 2A–C) and contralateral LC. The number and distribution of labeled cells (ipsi- vs. contralateral) was similar to that observed in previously published reports of LC projections to cortical targets using conventional retrograde tracer techniques (Simpson et al., 1997; Chandler et al., 2013; Plummer et al., 2020).

**FIGURE 3:**
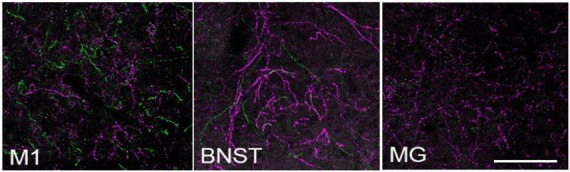
Locus coeruleus efferent fibers in TrAC-LC mice following CAV2-Cre injection in medial prefrontal cortex. tdTomato^+^ fibers (magenta) and EGFP^+^ fibers (green) are shown in primary motor cortex (M1), bed nuc stria terminalis (BNST), medial geniculate (MG). Note the paucity of labeled fibers in MG, i.e., axon collaterals of LC cells projecting to medial prefrontal cortex, scale bar = 100 μm. Adapted from Plummer et al. (2020).

**FIGURE 4:**
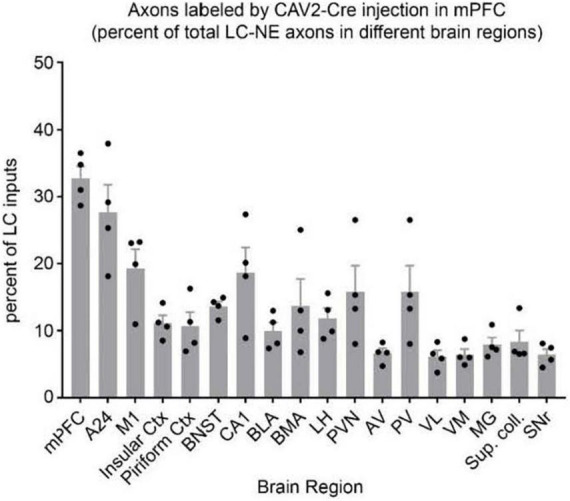
Distribution of axon collaterals from LC neurons projecting to mPFC. The bar graph (*n* = 4 mice) indicates percentage of LC-NE axons at each brain region that originate from EGFP + mPFC-projecting LC-NE neurons. LC-NE neurons are represented as percent of total LC-NE inputs (sum of EGFP + and tdTomato +) in select brain regions. mPFC, medial prefrontal cortex; A24, ventral anterior cingulate; insular ctx, insular cortex; M1, primary motor cortex; piriform ctx, piriform cortex; BNST, bed nucleus of the stria terminalis; CA1, area CA1 of the hippocampus; BLA, basolateral amygdala; BMA, basomedial amygdala; LH, lateral hypothalamus; PVN, paraventricular hypothalamic nuc; AV, anteroventral thalamic nuc; PV, paraventricular thalamic nuc; VL, ventrolateral thalamic nuc; VM, ventromedial thalamic nuc; MG, medial geniculate nuc; Sup. coll., superior colliculus; SNr, substantia nigra. Adapted from Plummer et al. (2020).

**FIGURE 5:**
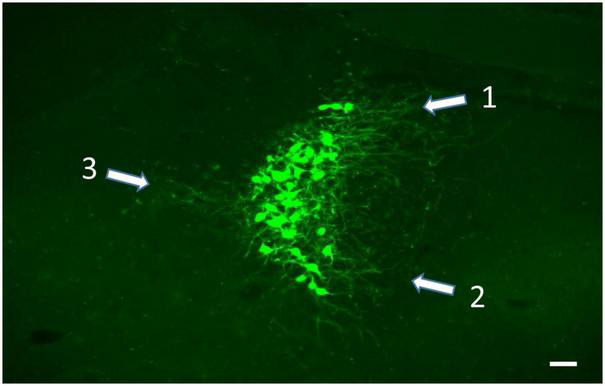
Soma and dendritic fields (white arrows) of LC neurons labeled from viral vector injection in the ipsilateral VL thalamus. Rather than extend evenly into the peri-coerulear surround, the dendrites from these cells are concentrated in the dorsomedial **(1)**, ventromedial **(2)**, and lateral **(3)** zones of the peri-coerulear space, scale bar = 100 μm.

In another preliminary analysis we found that although retrograde viral delivery of a recombinase into either medial prefrontal cortex or primary motor cortex produced similar numbers and distributions of retrogradely labeled LC neurons, the collateral network of axons from LC-mPFC projecting cells was not uniformly distributed across LC terminal fields (Figures 3, 4). Likewise, the distribution of axon collaterals from mPFC-projecting and M1 projecting LC-NE neurons differed from that of LC as a whole and from each other [see Figure 5 – (Plummer et al., 2020)].

In future work we might discover that the network of labeled axons from injections in different motor terminal fields is exclusive to motor circuitries across the CNS, a finding that would further emphasize the modular structure and selectivity of LC function in the context of motor control. We would also be able to determine the proportion of LC axons within a terminal field that arise from a specified cluster of retrogradely labeled neurons by comparing EGFP- vs. tdTomato-containing fibers within a given region (EGFP labeling/EGFP + tdTomato labeling).

Because motor pathways are crossed and because of the bi-lateral brainstem location of the LC, it will be important to pay strict attention to the laterality of labeled cells and the distribution of axon collaterals from these cells with respect to injection site and ipsi- vs. contralateral connectivity of motor pathways. This information will be critical for evaluating functional impact of these projections. Counter to expectations, it may be that axon collaterals from labeled LC cells are distributed without regard for terminal field modality, i.e., distribute uniformly and non-specifically to motor as well as non-motor areas of the brain. This observation would still be quite useful in validating or not the recently proposed modular design of the LC-NE system (Chandler et al., 2019).

### What is the intra-nuclear distribution of locus coeruleus cells that project to supraspinal motor circuits?

The distribution of cells within LC that have specified motor circuit projection targets is an important consideration that has received little attention relative to the distribution of LC cells which project to sensory and cognitive targets in the brain. In a previous study using conventional retrograde tracers (Simpson et al., 1997) we determined the intra-nuclear location and number of LC cells that project to the trigeminal somatosensory (whisker) pathway in rat. The study revealed a projection bias from LC to the trigeminal pathway that favored modulation of sensory signal transmission from one whisker pad to the opposite sensory cortex. As such, we were able to demonstate that LC projections to the trigeminal system maintain a functional alignment with the trajectory of sensory information from the periphery to the primary sensory cortex. Do LC-motor circuit projections display similar patterns of organization? The goal of future work is to establish or not an efferent topographic ordering of LC cells with respect to motor system targets that give rise to crossed and un-crossed control of motor functions.

**FIGURE 6:**
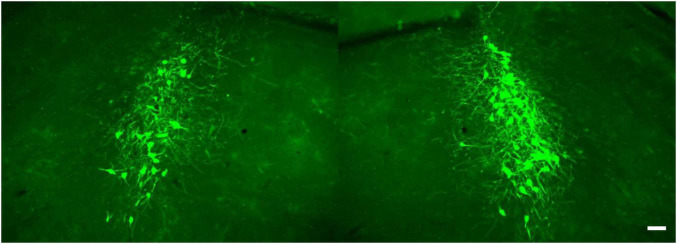
EGFP + NE-containing cells in the ipsi- (at **right**) and contralateral (at **left**) LC nucleus following unilateral injection of retrogradely transported viral vector in the cerebellar medial nuc, scale bar = 100 μm.

Results of ongoing studies indicate differential patterns of labeling following volumetric equivalent injections of viral vector in VL thalamus and cerebellar medial nuc (i.e., fastigial nuc in human), (Figure 6). Labeling in LC from both structures is bi-lateral but distributed with an ipsilateral bias (60% ipsi – 40% contra) relative to injection site. Althougth the rostro-caudal distribution of EGFP labeled cells is similar, output to the medial cerebellar nuc arises from a greater number of cells arranged in a tight cluster within the core of the nucleus. Neither of these distributions matches the pattern of labeling observed following volumetric equivalent injections of viral vector in prefrontal or motor cortices (see Figure 2). The LC projection to medial prefrontal and primary motor cortices arises from a scattered and primarily ipsilateral distribution of cells in the LC nucleus (Figure 2). We expected to find a similar scattered distribution of cell bodies in LC that project to various motor circuits. However the results from VL thalamus and medial cerebellar nuc injections (Figure 6) reveal a more bilateral and tightly clustered grouping of LC projection neurons to these regions in the central core of the nucleus (compare Figures 2, 6). This observation adds evidence in support of a heterogeneous organization of LC with respect to terminal field projections; in this case suggesting that different subsets of LC cells project to cortical vs. sub-cortical (VL thalamus) vs. cerebellar targets within the motor network.

Two issues are important when considering the results and interpretaton of retrograde labeling in the proposed study. First, the TrAC method alone does not allow for identification of sub-populations of LC soma that may have different projection targets. For example, although the intranuclear locations of LC-cerebellar cortex projection cells may appear to be uniquely distributed, these clusters of cells may overlap and intermingle with cells that project to other motor or non-motor terminal fields. Counterbalanced injections of conventional retrograde tracers, e.g., cholera toxin beta subunit - CTB, fluorescent dextrans, or retrogradely transported viral vectors in different LC terminal fields would be capable of revealing within the same animal the intranuclear locations of LC soma with different terminal field targets. The second consideration is tissue tropism for viral vectors, i.e., the capability of viruses to infect different tissues. Three neuronal cell types have been described in rodent LC based upon morphology of the cell soma; multipolar, fusiform, and round (Loughlin et al., 1986). Although only speculative, we assume that viral vectors used in the proposed study have equal tropism for axons emanating from these different LC cell types.

### Do the dendritic arbors of locus coeruleus-motor circuit projection neurons have a preferred distribution within the peri-coerulear space?

In rodents the LC consists of a dense core of cell bodies surrounded by dendritic processes that extend at least 75–100 μm and in some cases 500–600 μm into the neuropil from the border of the nucleus (Cintra et al., 1982; Shipley et al., 1996). These processes form a halo around the nucleus in an area referred to as the peri-coeruelar space, a region that is sparsely populated by cell bodies from other brain structures (Cintra et al., 1982; Shipley et al., 1996). In rat there are three primary zones within the peri-coerulear space into which dendrites arising from LC neurons project; (1) rostral medial, (2) caudal medial, and (3) dorsal lateral with respect to the core of the nucleus (Van Bockstaele et al., 1996a,b, 1999a,1999b). Inputs to these zones are from central amygdala, bed nucleus of stria terminalis, paraventricular hypothalamus, nucleus tractus solitarius, peri-acqueductal gray, and prefrontal cortex (Van Bockstaele et al., 1996a, b, 1998, 1999a, 1999b, 2001; Jodo et al., 1998; Reyes et al., 2011). Light and EM studies have determined that inputs from these structures have preferred targets among these three primary dendritic fields of LC cells (Van Bockstaele et al., 1996a,^1998^, 1999a,1999b). For example, the dorsal lateral peri-coerulear space is targeted by afferents from the central amygdala and cardiovascular region of the nucleus tractus solitarius (Van Bockstaele et al., 1999a). EM studies have further determined that afferents to this zone of the peri-coerulear space make monosynaptic connections (excitatory and inhibitory) with LC dendrites and as such are capable of regulating LC output, presumably under conditions where limbic and autonomic system signals converge to influence NE release in the LC terminal fields (Van Bockstaele et al., 1999a,1996b,^2001^). Overall, these studies suggest that inputs to LC are non-overlapping and topographically ordered. *Despite these findings there have been no studies capable of determining the distribution of dendrites from LC neurons with known efferent targets.* By way of retrograde viral labeling TrAC allows for identification of subsets of LC neurons with defined projection targets and visualization of their dendritic arbors. An important goal of future studies is to compare the peri-coerulear dendritic fields of LC-motor vs. LC-non-motor circuit projection neurons. We postulate that relative to non-motor LC projection cells, LC-motor network projection neurons have dendritic fields with unique patterns of distribution within the peri-coerulear space.

Because of the clarity with which retrogradely labeled LC cells expressing EGFP are visualized, we have started to use TrAC to examine the dendritic fields of LC-motor circuit projection neurons. As shown in Figure 5 the dendritic arbors of cells that project to VL thalamus are concentrated in three zones of the peri-coerulear space; dorsal medial, ventral medial, and lateral. By contrast cells projecting to medial cerebellar nucleus are restricted to dorsal medial and ventral medial peri-coerulear zones (data not shown). The distribution of dendrites arising from LC cells with known efferent targets is an open question that must be answered to further pursue the idea that the input-output relationships of LC are not random but rather maintain a selective organization with respect to the function of LC-NE terminal fields. In the case at hand, the focus should be on LC projections to motor centers in the brain. The distribution of dendrites arising from LC-motor projection neurons can be matched against the input fields from prefrontal cortical, limbic, and autonomic structures that are afferent to sub-regions of the peri-coerulear space (Van Bockstaele et al., 1996a,^1998^, 1999a,1999b,^2001^; Reyes et al., 2011). The location and size of LC neuron dendritic fields determines the region from which afferent inputs can be sampled and therefore identifies potential source(s) of afferent regulation of LC activity. For example, because the projections from prefrontal cortex and central amygdala terminate discretely in medial vs. dorsal lateral zones of the peri-coerulear space, respectively (Arnsten and Goldman-Rakic, 1984; Van Bockstaele et al., 1999b), it should be possible to differentiate the dendritic fields and potential synaptic receptive zones of LC-motor circuit projection neurons in terms of these two areas. Based upon our preliminary observations, we predict a restricted distribution of the dendritic arbors of LC-motor circuit projection neurons within the peri-coerulear space. Alternatively, LC-motor projection neuron dendritic fields may overlap completely with known afferent input fields and therefore not exhibit any specificity with respect to these inputs. Additional questions can be considered. Is there a differential distribution of dendritic arbors arising from LC cells that project to different motor targets? Are there differences in the distribution of dendritic fields arising from motor vs. sensory (dorsal lateral geniculate or visual cortex) circuit projection neurons.

### Do locus coeruleus-motor circuit projection neurons exhibit unique electrophysiological properties relative to locus coeruleus cells that project to non-motor circuits?

**FIGURE 7:**
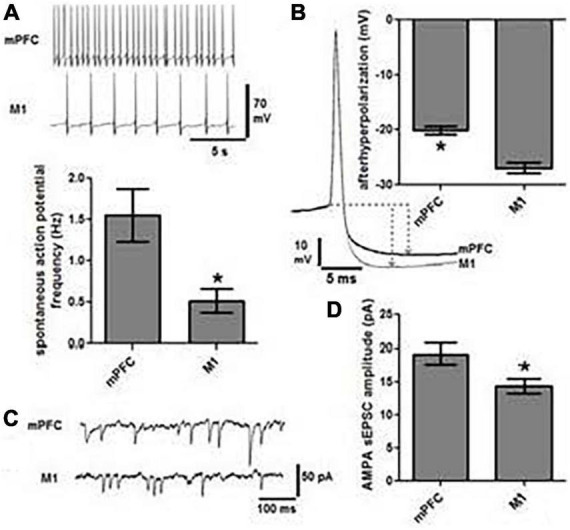
Locus coeruleus cells projecting to mPFC are physiologically distinct from those projecting to M1. **(A)** Representative traces of spontaneous action potentials indicate that cells projecting to mPFC (*n* = 19) fire three-fold faster than those projecting to M1 (*n* = 19, *p < 0.05). **(B)** The magnitude of afterhyperpolarization (AHP), as determined by the difference in voltage between action potential threshold and the lowest point of the AHP (dashed lines and arrows), was significantly lower in mPFC projection cells than those terminating in M1 (*p < 0.05). **(C)** Representative traces of AMPA mediated spontaneous excitatory post synaptic current (sEPSC) and the graph in panel **(D)** indicate that the amplitude of sEPSCs was significantly greater in mPFC vs. M1 projection cells (*p < 0.05).

Identification of retrogradely labeled LC neurons in *ex vivo* brain slices provides a means for performing whole cell patch clamp recordings of the neurons that project to motor circuits. This approach can be used to determine intrinsic membrane properties, spontaneous discharge, and synaptic response properties of subsets of LC projection cells. Published studies from our laboratory indicate that LC-medial prefrontal cortex projecting neurons have higher rates of basal discharge (Figure 7A), shallower afterhyperpolarization (Figure 7B), and higher amplitude of AMPA-mediated sEPSCs (Figures 7C,D) relative to LC-primary motor cortex projection neurons (Chandler et al., 2014a). Such an arrangement may have considerable behavioral significance insofar as LC-prefrontal cortex projection neurons appear to have lower thresholds of activation and higher basal discharge rates than LC-primary motor cortex projection cells, thus prompting greater NE release and more robust noradrenergic modulatory actions in decision-making circuits relative to movement generating circuits. At basal levels of LC output, this dynamic would facilitate focused and flexible attention, decision-making and execution of behaviors guided by the prefrontal cortex. Furthermore, because noradrenergic modulation follows an inverted-U dose-response function (Berridge and Waterhouse, 2003; Aston-Jones and Cohen, 2005a,b; Devilbiss et al., 2006; Moxon et al., 2007), we would expect increasing LC output to achieve optimal NE modulatory effects in motor cortex while modulatory actions in prefrontal cortex are waning. This asynchronous mode of operation would facilitate transitions between exploitation of successful behavioral strategies and pursuit of alternatives to meet new behavioral contingencies, as suggested by the theoretical constructs proposed by Aston-Jones and Cohen (Aston-Jones and Cohen, 2005a,b).

Because LC projection targets are variously engaged in cognition, affect, sensation, and movement we postulate that the physiological properties of the LC neurons modulating each of these modalities are distinct. The demonstration that different classes of LC neurons projecting to defined targets have unique electrophysiological properties would provide strong support for the emerging hypothesis that the LC-NE system can differentially and asynchronously modulate activity across different modality-specific terminal fields. Note, this result has already been demonstrated for the VTA-dopamine system (Lammel et al., 2008). We believe that LC-motor circuit projection cells will display similar firing patterns and discharge frequencies in response to current injection, and likewise exhibit similar synaptic responses. These properties would differ from those expressed by cells projecting to non-motor regions of the brain; e.g., sensory targets of LC such as the dorsal lateral geniculate nucleus. Different basal firing rates and excitability of LC projection neurons would translate to differential NE release and NE action in LC terminal fields. For example, a lower threshold influence of LC output on cognitive and/or sensory processes and a higher threshold influence on motor functions might lead to LC-NE modulation of sensory signal processing and decision-making operations before LC-NE regulation of networks responsible for goal-directed movement – i.e., *“sensing and planning before doing.”* This outcome would provide evidence for segregation of function within the LC-NE transmitter system and as such would establish the foundation for an entirely new theoretical construct for explaining the role of LC in brain function and behavior.

### Does selective activation of locus coeruleus-motor network projection neurons improve performance in tests of coordination, balance, and forelimb dexterity?

Locus coeruleus activation, local administration of NE, or pharmacologic elevation of NE levels in the brain enhances sensory signal processing at cellular, circuit and network levels and improves performance in sensory guided behaviors(Waterhouse and Navarra, 2019). The LC-NE system has been shown to exert similar influences on cognitive circuitries and executive functions (Bouret and Sara, 2004; Sara and Bouret, 2012; Bari et al., 2020; Carlson et al., 2021). By contrast similar studies have not probed the impact of the LC-NE system on motor circuit operations and motor behavior. Early work showed that noradrenergic pharmacotherapy, adrenal transplantation, and direct infusion of NE into motor regions of the CNS have some value in promoting functional recovery after stroke and traumatic brain injury (Feeney et al., 1982, 1993) but the moment-to-moment regulation of motor network function and movement control by the LC-NE system has not been studied in normal animals. A strategy to address this question is to assess transgenic mouse performance in well-established rodent motor tasks; before, during and after chemogenetic or optogenetic activation (or inactivation) of LC cells with known projections to primary motor cortex. This approach would increase (or decrease) release of NE in not only primary motor cortex but also the motor related terminal fields that receive axon collateral distributions from these cells.

To enable control of the output of LC cells that selectively project to primary motor cortex, mice would be manipulated to express designer receptors exclusively activated by designer drugs (DREADDS) in LC-primary motor cortex projection cells. This would allow for activation or suppression of these cells by the chemogenetic actuator deschloroclozapine, while mice are performing motor skill tests. Activation or suppression of visual cortex-projecting LC cells during motor skill tests would serve as controls. Using a similar strategy animals could be tested in motor tasks before and during optogenetic activation of targeted LC cells. Many tests of motor function can be employed to examine the impact of LC output on motor behavior. For example, the accelerating rotorod test assesses motor coordination by measuring latency to fall from an accelerating rotating drum (Brooks and Dunnett, 2009). The balance beam test assesses the animal’s ability to maintain balance while traversing an increasingly narrow plank (Brooks and Dunnett, 2009; Luong et al., 2011). The staircase test measures skilled reaching using a baited descending staircase on either side of a center beam (Brooks and Dunnett, 2009). The number of food pellets retrieved measures bilateral skilled reaching. Sex as a variable can also be added to each of these motor behavioral protocols.

These types of experiments could determine if performance of motor tasks can be improved or diminished, respectively, by selective activation or inactivation of LC-primary motor cortex projection cells. Because of ceiling effects in performance of any or all tasks, suppression of LC output *via* inhibitory DREADD and decreased task performance might be a better indicator of LC influence on motor behavior. We would not expect changes in motor performance in littermate controls not expressing DREADDS or animals where DREADDS are expressed in LC-cells projecting to visual cortex. The tasks described above, i.e., rotorod, balance beam, and staircase test; are used routinely in mice to evaluate motor coordination, balance, and manual dexterity, respectively (Brooks and Dunnett, 2009; Luong et al., 2011). In particular, these tasks have been used to assess the degree of motor dysfunction and recovery in mouse models of head trauma (Feeney et al., 1982; Gulinello et al., 2008), stroke (Bouet et al., 2007), and neurodegenerative disease (Carter et al., 1999; Gulinello et al., 2008; Southwell et al., 2009).

## Implications for disease, injury, or age-related motor dysfunction

The experimental strategies described here have the potential to address major unanswered questions about the structure of the LC-NE transmitter system and its potential role in CNS control of balance, posture, and voluntary and reflexive movements in health and disease. The working hypothesis is that output from a sub-domain of LC is capable of coordinately and simultaneously regulating operations across a distributed network of motor circuits with the end-result of facilitating motor responses according to behavioral demands placed on the organism. For example, in healthy animals, as output from LC waxes and wanes across the waking cycle, we might expect moment-to-moment fluctuations in reaction time, motor coordination, and speed and accuracy of goal-directed movements. Such effects could occur independently or in conjunction with LC-mediated modulation of sensory signal processing and cognitive function depending on the modular or global mode of LC activation. Motor learning might also be affected by changes in LC output. Chemo- and optogenetic approaches have already been used to probe the effects of LC output on sensory signal processing, sensory guided behaviors, and executive functions (Kane et al., 2017; Cope et al., 2019; Glennon et al., 2019; Carlson et al., 2021); and one recent study has used both global and local activation of NE release to rescue a motor learning deficit in a mouse model of autism (Yin et al., 2021). However, a criticism of this published work is the use of either global or local activation/inactivation (electrical, opto- or chemogenetic) of LC or LC fibers to prompt widespread, indiscriminate or narrowly focused release of NE in LC-NE terminal fields. While these studies have been useful for demonstrating the potential brain-wide or local actions of LC activation in waking animals, they do not provide the means to isolate LC-NE actions on ensembles of circuits in modality-specific networks such as those that exist in the motor system. The use of TrAC to label the collateral axon networks of LC-motor circuit projection cells will provide the roadmap and rationale for this more targeted approach. While initial studies can be conducted in normal animals to assess the role of LC output in routine motor tasks, future work can evaluate the benefits of activating motor circuit projections of the LC-NE system as a means of restoring balance, range, rhythm, and rate of movement in aged animals and restoring motor function in animal models of disease (Carter et al., 1999; Von Coelln et al., 2004; Liu et al., 2013), neurodevelopmental disorders (Piochon et al., 2014; Del Pino et al., 2018; Yin et al., 2021), stroke (Bouet et al., 2007), and TBI (Feeney et al., 1993; Osier and Dixon, 2016).

## Data availability statement

The original contributions presented in this study are included in the article/supplementary material, further inquiries can be directed to the corresponding author.

## Ethics statement

The animal study was reviewed and approved by the Rowan University IACUC.

## Author contributions

BW conceptualized and wrote the report with editorial comment from HP, NP, PJ, and DC. NP and PJ developed the TrAC methodology and contributed to the preparation of Figure 1. BW, NP, PJ, and DC developed the TrAC experimental strategies. DC, NP, and PJ contributed to the data collection and preparation of Figures 2–4. HP contributed to the data collection and preparation of Figures 5, 6. DC contributed to the data collection and preparation of Figure 7. All authors contributed to the article and approved the submitted version.
